# The 2018 UK NHS Digital annual report on the Improving Access to Psychological Therapies programme: a brief commentary

**DOI:** 10.1186/s12888-019-2235-z

**Published:** 2019-08-14

**Authors:** Naomi Petra Moller, Gemma Ryans, Jasmine Rollings, Michael Barkham

**Affiliations:** 1 The British Association for Counselling and Psychotherapy, BACP House, 15 St John’s Business Park, Lutterworth, LE17 4HB UK; 2 School of Psychology, The Open University, Walton Hall, Milton Keynes, MK7 6AA UK; 3 Department of Psychology, Centre for Comparative and Evolutionary Psychology, University of Portsmouth, University House, Winston Churchill Avenue, Portsmouth, PO1 2UP UK; 4 Clinical Psychology Unit, Department of Psychology, The University of Sheffield, Sheffield, S10 2TP UK

**Keywords:** Improving access to psychological therapies, Cognitive Behavioural therapy, Counselling for depression

## Abstract

This commentary examines publicly available information on 2017–2018 outcomes in the UK government’s Improving Access to Psychological Therapies (IAPT) programme, a National Health Service (NHS) primary care mental health programme in England. In that year there were 1.4 million referrals into IAPT and over 500,000 people completed a course of treatment. The IAPT database collects routine session-by-session outcome monitoring data for this population, including outcomes for depression and anxiety in a stepped care model which includes a range of psychological therapies, among them Cognitive Behavioural Therapy (CBT) and Person-centred Experiential Therapy, known in the IAPT programme as Counselling for Depression (CfD).

In 2017–18, 32% of all referrals were for anxiety and stress disorders, 26% for depression, and 35% were unspecified. The definition of treatment completion is receipt of 2 sessions or more and on this basis 60% of all referrals in 2017–18 did not complete treatment, predominantly because they failed to attend the initial appointment, or ended after only one session. Four years of data on outcomes for CBT and CfD suggests these therapies are broadly comparable in terms of both recovery rate and average number of sessions, though the number of referrals to each therapy varied widely. Data on treatment choice and satisfaction was favourable but there were issues with low return rates and invalid data. Information on outcomes for ethnicity, sexual orientation, disability and religion, as well as a measure of local economic deprivation, indicate lower outcomes for a number of patient groups. Data on employment status outcomes suggest little overall change, including for the category of those on benefits payments.

The data published alongside the annual IAPT reports mean there is an increasing amount of information in the public domain about IAPT performance, but it is time consuming to extract and evaluate. This report highlights a number of points of concern which suggest the need for improvement on multiple axes. We suggest that improved researcher access to the huge IAPT dataset can allow for more detailed evaluations of IAPT that can inform policy/decision-making to improve outcomes for clients.

## Background

This article delves into publicly available information on the Improving Access to Psychological Therapies (IAPT) programme to examine IAPT performance [1]. The UK government’s IAPT initiative is a funded programme for England aimed at delivering evidence-based psychological therapies – primarily CBT – for depression and anxiety in a stepped care model [2]. All patients complete a minimum dataset at each session including the Patient Health Questionnaire-9 (PHQ-9) and Generalised Anxiety Disorder-7 (GAD-7). Data is submitted to NHS Digital and an annual report is made public. The 2017–2018 annual IAPT report was published in November 2018 [3], followed by supplementary reports in 2019 [4]; the presentation of data is similar to prior IAPT reports [5–7], although since the first IAPT annual report (for 2012–2013) there have been annual changes in what data is presented and in the most recent report fewer Excel tables are provided, with data being presented for the first time through an interactive dashboard.

The IAPT programme has been described as “England’s mental health experiment” [8] and it is important to consider the outcomes of this experiment since 2012. The current article focuses on key results from 2017 to 2018 but includes, where relevant, results from the prior three IAPT reports.

## Main text

### Most common presenting issues

In 2017–18, out of the 1,439,957 referrals, 35% (*N* = 498,060) were unspecified (“not stated, not known, invalid”), 26% (*N* = 375,001) were for depression and 32% (*N* = 467,911) for anxiety and stress disorders, a category that included Generalised Anxiety Disorder (GAD), mixed anxiety and depression, Agoraphobia, Obsessive Compulsive Disorder, Panic Disorder, Post-Traumatic Stress Disorder, Social and specific phobias and other anxiety or stress related disorders [3]. Thus, while depression and anxiety comprise the most common issues referred to IAPT, there is a lack of information about more than one-third of the referrals.

### Declining offer of treatment, completing treatment, and dropout

Data shows that in 2017–18, of the 1,376,920 referrals that ended (e.g. completed treatment in 2017–2018), 40.3% (*N* = 517,942) completed a course of treatment, where treatment completion was defined as having a minimum of 2 sessions [3]. Of the rest, 29% (*N* = 398,443) ended before being seen by the service (e.g. chose not to attend the first appointment), 2% (*N* = 28,733) were seen by the service but not treated (e.g. were found to be unsuitable for the service) and 29% (*N* = 395,035) ended after having had only one appointment (e.g. dropped out of treatment) [3]. The figures mean that 60% of all the referrals that ended in 2017–2018 did not ‘complete’ treatment; given the definition of treatment completion is 2 sessions or more, the cited figures likely mask higher rates of dropout.

### Outcomes for CBT versus CfD

CBT and CfD (previously termed ‘counselling’ in the IAPT dataset) are both one of a range of ‘High Intensity Treatments’ offered at Step 3 in IAPT. Historically the CfD outcome data has pooled outcomes from CfD (Person-centred Experiential Therapy, PCET) and generic counselling (since 2018 all CfD practioners are required to be PCET trained [9]). Recovery is defined in IAPT as moving from caseness at the start of treatment out of caseness at the end of treatment [3]. For the PHQ-9, which is an assessment of the severity of depression, the cut-off score for caseness is 10 and for the GAD-7 it is 8. Table 1 presents data on the number of referrals or courses of therapy, average number of treatment sessions, recovery rate, and recovery rate per session [4–7]. It should be noted that recovery rates for 2017–2018 are not directly comparable with prior figures due to a focus in this year on outcomes of individual courses of therapy versus an individual’s outcome from all IAPT interventions prior to discharge. The data in Table 1 suggests recovery rates have improved over time and that there is very little difference between CBT and Counselling/CfD in terms of recovery rate or number of sessions in terms of overall outcomes, depression or the broad category of anxiety and stress-related disorders. In 2017–2018 the mean pre-therapy PHQ-9 score was 14.7 for CBT and 15.0 for CfD while the mean pre-therapy GAD-7 score was 13.6 for CBT and 13.1 for CfD, suggesting comparable levels of psychological distress in the populations referred to these therapies [10]. But marked differences are evident in the number of courses of therapy/referrals for CfD and CBT, with more than twice as many courses of CBT than CfD in 2017–2018. The small differences in overall recovery rates present a challenge to the view that CBT is the preferred treatment of choice for depression and for anxiety when delivered in routine NHS settings. The average higher recovery rate per session also suggests that counselling/CfD may offer an additional advantage in terms of efficiency.

**Table 1:** IAPT outcomes for CBT and counselling/counselling for depression

Year	Intervention	Courses of therapy	Average number of sessions	Recovery rate (%)	Average recovery rate per session (%)
Overall outcomes					
2017–2018	CBT	176,166	7.4	47.4	6.4
	Counselling (CfD)	74,106	6.3	47.0	7.5
Year	Intervention	Number of referrals for specific disorder	Average number of sessions	Recovery rate (%)	Average recovery rate per session (%)
Depression					
2016–17	CBT	45,746	5.9	47.3	8.0
	Counselling (CfD)	29,265	5.6	50.2	9.0
2015–16	CBT	35,589	5.8	45.9	7.9
	Counselling (CfD)	20,011	5.3	47.6	9.0
2014–15	CBT	28,350	5.9	44.1	7.5
	Counselling (CfD)	14,994	5.2	45.2	8.7
Anxiety					
2016–17	CBT	100,965	6.3	50.5	8.0
	Counselling (CfD)	28,988	5.2	48.4	9.3
2015–16	CBT	84,155	6.4	49.0	7.7
	Counselling (CfD)	20,922	5.2	46.7	9.0
2014–15	CBT	66,799	6.3	47.5	7.5
	Counselling (CfD)	15,991	5.0	44.9	9.0

Note: average recovery rate per session is calculated as recovery rate (as a %) divided by average number of sessions

### Treatment choice and satisfaction

In terms of treatment choice, 19% (*N* = 192,414) of those who entered treatment in 2017–2018 (1.01 million), completed the assessment questionnaire [3]. Out of these, 71% of patients indicated they were offered a choice of treatments, 60% reported that they had a treatment preference, and 60% that they were offered their treatment preference [3]. However, for the three choice items, about 26% of the responses were coded as ‘invalid’. In 2017–18, of the 554,709 patients who completed a course of treatment, 22% (*N* = 121,512) completed the five patient treatment questions [3]. Averaging across the questions, over 80% of patients selected ‘at all times’ or ‘most of the time’ when responding to the five (positively keyed) items inquiring about their experience of services [3]. Yet, for both treatment choice and satisfaction, low response rates, issues with invalid data and the tendency for patients to respond positively to these questions, create uncertainty about the findings.

### Outcomes related to client diversity

The 2017–18 IAPT report presents information on referrals and outcomes for: age, gender, ethnicity, sexual orientation, disability and religion, as well as Indices of Deprivation, a measure of local economic deprivation [3]. For example, data suggests a linear correlation between level of deprivation and (1) referrals to IAPT (positive correlation) and (2) recovery rates (negative correlation). For patients in the most and least deprived areas, there is a 17% difference in the recovery rate: 58.1% for least derived area, 41.0% for most deprived area, versus the overall recovery rate reported for 2017–2018 of 50.8%. For religion, there is a 14% difference in recovery rates between Christians (recovery rate 54.5%) and Muslims (40.3%); in terms of referrals Muslims are the largest non-Christian religious group from those who profess a religion. Non-disabled people have a recovery rate of 53.6%; the best recovery rate for individuals with a disability is for those with hearing disability (50.8%) but those reporting speech, sight, physical health conditions and learning disabilities have recovery rates ranging from 42 to 48%, while those with other forms of disability, including mobility and behavioural and emotional issues, have recovery rates under 40%. Heterosexuals have a recovery rate of 51.8%; gay/lesbian people, 47.9%, and bi-sexual people 41.4–10% lower than the heterosexual recovery rate. The recovery rate for white people is 51.7%; for Asian/Asian British, Black/Black British and mixed ethnicity groups it is over 5% less. This evidence of unequal outcomes for those with characteristics protected by UK statute [11] is clearly of concern.

### Change in employment status

A key argument for the IAPT programme was that greater access to treatment would reduce unemployment and sickness benefits claims [1]. The 2017–18 report presents data on employment status at the beginning and end of treatment [3]. The report includes a variety of employment categories, including a number for those not actively seeking work, such as retired people, homemakers and students. While the data suggests changes by individuals *across* categories, there appears to be little shift in overall numbers for the key categories. For example; *Employed*: Start of treatment, 316,604; end of treatment, 302,746; *Unemployed and seeking work*: Start of Treatment, 54,580; End of Treatment, 49,803; *Long-term sick or disabled or in receipt of benefits*; Start of Treatment, 43,275; End of Treatment, 43,671. The category for those on benefits payments includes those on incapacity benefit, income support, or both, as well as those on employment and support allowance.

## Conclusions

Although there is an increasing amount of information in the public domain about IAPT performance in the annual reports, there is a considerable burden on the reader to extract and then construct a report such as detailed in this Commentary. There are a number of points of concern. The data on treatment choice and satisfaction with services is a positive indicator of IAPT’s acceptability to patients but doubts are caused by the low return rates, issues with invalid data and the propensity for patients to respond positively to these questions. Further, the data also indicates that 60% of those referred to IAPT do not complete the initial 2 appointments; given the low threshold for ‘completing treatment’, the dropout rate is potentially significantly higher. One question is whether the ‘right’ patients are being referred to IAPT; the service is aimed at people with depression and anxiety yet one third of patients had unspecified presenting issues which may mean that inappropriate referrals are being made. The data on outcomes for CBT and CfD also challenges assumptions – implicit in disproportionate referrals to each modality - in IAPT about the relative merits of the two most commonly offered therapies in that the outcomes of these therapies were broadly similar. There was also some evidence of greater efficiency for CfD. It is known that client preferences impact both overall recovery and treatment completion [12] and IAPT reports data on whether clients are offered a choice of interventions and whether they have preferences, yet it is unknown from the currently available public IAPT reports how outcomes and client preferences are related. Given that the NHS has a legal requirement to reduce inequalities in health outcomes [13], it is concerning that outcomes for a number of patient populations are consistently poorer. Equally it is important that there is very little change in the numbers related to benefits and employment status, although this was originally a primary aim for the IAPT programme.

The many achievements of the IAPT programme, particularly its value in bringing NHS mental health treatment to increasing numbers of people in England, cannot be underestimated. Given its status as the largest social experiment in the psychological therapies and the invaluable data collected, we would encourage improving access to such data so that it can be shared more widely in order to inform policy/decision-making to improve outcomes for clients and to enhance knowledge of the psychological therapies. This would be timely given increasing interest internationally in how to deliver quality, publicly funded primary care mental health services [14] and the arguments that the evidence base for the effectiveness of psychological treatments for mental health within primary care needs to be strengthened [15].

## Data Availability

Hyperlinks to publicly archived datasets analysed during the study are provided in the references.

## Abbreviations

CBT: Cognitive Behavioural Therapy
CfD: Counselling for Depression
GAD-7: Anxiety screening instrument, Generalised Anxiety Disorder-7
IAPT: Improving Access to Psychological Therapies
NHS: UK’s National Health Service
PCET: Person-Centred Experiential Therapy
PHQ-9: Depression screening instrument, Patient Health Questionnaire-9
