# Correction to: The 2018 UK NHS Digital annual report on the Improving Access to Psychological Therapies programme: a brief commentary

**DOI:** 10.1186/s12888-019-2277-2

**Published:** 2019-10-17

**Authors:** Naomi Petra Moller, Gemma Ryan, Jasmine Rollings, Michael Barkham

**Affiliations:** 1 The British Association for Counselling and Psychotherapy, BACP House, 15, St John’s Business Park, Lutterworth, LE17 4HB UK; 2 School of Psychology, The Open University, Walton Hall, Milton Keynes, MK7 6AA UK; 3 Department of Psychology, Centre for Comparative and Evolutionary Psychology, University of Portsmouth, University House, Winston Churchill Avenue, Portsmouth, PO1 2UP UK; 4 Clinical Psychology Unit, Department of Psychology, The University of Sheffield, Sheffield, S10 2TP UK

**Correction to: BMC Psychiatry**

**https://doi.org/10.1186/s12888-019-2235-z**

Following publication of the original article [1], we have been notified that one of the authors’ names is spelled incorrectly. In this Correction the incorrect and correct author name are shown.

Originally the author name has been published as:

- Gemma Ryans

The correct author name is:

- Gemma Ryan

The original article has been corrected.
